# The Law Relating to Lunatics and Persons of Unsound Mind.—III

**Published:** 1903-03-28

**Authors:** J. E. R. Stephens

**Affiliations:** Barrister-at-Law, Author of "Digest of Public Health Cases"


### HOSPITAL ADMINISTRATION.

### CONSTRUCTION AND ECONOMICS.

### THE LAW RELATING TO LUNATICS AND PERSONS OF UNSOUND MIND.—III.

By J. E. R. Stephens, Barrister-at-Law, Author of "Digest of Public Health Cases."

(*Continued from page* 361.)

Jurisdiction in Lunacy.

The custody of idiots and lunatics has from a very early period in England been vested by the law in the Crown. The statute *De Praerogativa Regis* (17 Edw. II. c.c. 9 and 10) provided that the King (*a*) should have the custody of the lands of idiots, taking the profits of them without waste or destruction, subject to the conditions that he should supply the idiot with necessaries and restore the lands to his heirs after his death; and (*b*) in the case of lunatics should see that their lands and tenements were safely kept without waste and destruction, that they and their household were competently maintained out of "the issues of the same," and that the residue was to be kept for their use on recovery, or, if they died, distributed for their souls by the advice of the Ordinary. It will be noticed that under this old statute the idiot and the lunatic occupy different positions. In the case of the idiot, the King took the profits of the estate to his own use; in that of the lunatic he was merely a trustee. The distinction soon became, however, of little importance, as juries returned verdicts of *non compos mentis*, instead of idiocy, wherever they could; and it seems that after the Revolution it became the practice of the Crown to grant the surplus profits of the estate of an idiot to some of his own family.

The Crown has, from at least the sixteenth century, exercised its control over the persons and property of lunatics by delegates, first by the Lord Chancellor, then by the Court of Wards (32 Henry VIII. c. 46), and again by the Lord Chancellor, after the Court of Wards was abolished. But it was not as head of the Court of Chancery that the Lord Chancellor exercised the jurisdiction in lunacy, but as the representative and delegate of the Crown.

An inquisition may be ordered on a report of the Commissioners in Lunacy to the Lord Chancellor. This, however, is usually directed to be held on the application of a near relative of the alleged lunatic, and, indeed, the petition for an inquisition by which the proceedings are commenced ought to be presented by the nearest relative. It may, however, if the circumstances render it necessary, be preferred by an executor or trustee, a tenant, a creditor, or the Attorney-General. The petition is supported by affidavits by medical men, preferably unconnected with lunatic asylums, and others (if necessary) setting forth the facts that show the incapacity of the alleged lunatic.

Under section 116 of the Lunacy Act, 1890, summary proceedings may be taken without inquisition—for example where the property does not exceed £2,000 in value or yield an annual income of more than £100. The proceedings by way of inquisition, which are the oldest form of statutory interference with the insane, are in ordinary cases very costly; consequently they can only be taken in the case of persons of considerable wealth, and should, moreover, never be resorted to, even in such cases, unless the insanity is either permanent or grave inconvenience and difficulty will be caused in the management of important business or property by its existence for any time which is likely to prove substantial. The great bulk of the insane, however, do not belong to the wealthy class whose friends are able to indulge in the expensive luxury of getting them found lunatic by inquisition. Many of them are drawn from the middle classes, who are either possessed of small incomes, with which they are just able to maintain themselves, or else are the recipients of assistance from charities, which thus preserves them from falling into the pauper classes. Such persons, for the most part, fall under the operation of, and are detained under orders made under, the provisions of the Lunacy Act, 1890. And the numerical majority of the insane actually belong to the pauper class.

Removal of Insane Persons by Summary Reception Orders.

Private patients, whom it becomes necessary to place under the restraint authorised by the lunacy laws, may, in acute cases which call for immediate treatment, be made the subject of an "Urgency Order." In ordinary cases, however, private patients must be made the subject of a "Reception Order," obtained upon petition to a "judicial authority."

In cases of urgency where it is expedient, either for the welfare of a person (not a pauper) alleged to be a lunatic, or for the public safety, that the alleged lunatic should be forthwith placed under care and treatment, he may be received and detained in an institution for lunatics, or as a single patient upon an urgency order, made (if possible) by the husband or wife or by a relative of the alleged lunatic, accompanied by one medical certificate (section 11 of the Lunacy Act, 1890). There is "urgency" within the meaning of the section only where instant intervention is "expedient, either for the welfare of the alleged lunatic or for the public safety." The procedure is not to be resorted to in cases not falling under one or other of these categories. A pauper lunatic may in urgent cases be removed to a workhouse by a constable, relieving officer, overseer, or by order of a justice. The urgency order is a sufficient authority for the reception and detention if accompanied by the prescribed medical certificate and a statement of particulars. He is to be detained for seven days, or if a petition for a reception order is pending, then till it is disposed of. Apart from any statutory authority the detention of a lunatic is justifiable if necessary for his protection or that of others. Wilful misstatement of material fact in an urgency order is a misdemeanour. If not related to the patient, the person signing the urgency order must state the reason why the order is not signed by a relation, his or her connection with the patient, and the circumstances under which he or she signs. The order may be signed before or after the certificate. The medical certificate must contain a statement signed by the person signing the certificate that it is expedient for this alleged lunatic to be placed under care and treatment forthwith. This is in addition to the statement of particulars signed by the person making the reception order. It must appear by the medical certificate that the certifying practitioner has personally examined the alleged lunatic not more than two clear days before his reception, otherwise the lunatic is not to be received.

Every constable, relieving officer, or overseer of a parish, who has knowledge that any person within the district or parish of the constable, relieving officer, or overseer, who is not a pauper and not wandering at large, is deemed to be a lunatic and is not under proper care and control, or is cruelly treated or neglected by any relative or other person having the care or charge of them, shall within three days after obtaining such knowledge give information thereof upon oath to a justice. Any other person having such knowledge has a right at any time to lay a similar information before a justice qualified to act under this section. It is the duty of the relieving officer to move under this section, even if he knows that although the lunatic is not a pauper, the expense of sending him to an asylum and of his maintenance there will probably fall upon the rates. The justice, upon the information on oath of any person whomsoever, may himself visit the alleged lunatic, and shall authorise any two medical practitioners whom he thinks fit to visit and examine the alleged lunatic and to certify their opinion as to his mental state. If upon the certificates of the medical practitioners who examine the alleged lunatic, or after such other or further inquiry as the justice thinks necessary he is satisfied that the alleged lunatic is a lunatic, and is not under proper care and control, or is cruelly treated or neglected, the justice may by order direct the lunatic to be received and detained in an institution for lunatics to which, if a pauper, he might be sent, and the constable, relieving officer, or overseer upon whose information the order has been made, or any constable whom the justice may require so to do, shall forthwith convey the lunatic to the institution named in the order. Under section 14 of the Lunacy Act, 1890, every medical officer of a union who has knowledge that a pauper resident within the district of the officer is, or is deemed to be, a lunatic and a proper person to be sent to an asylum, shall, within three days after obtaining such knowledge, give notice thereof in writing to the relieving officer of the district, or, if there is no such officer, to an overseer of the parish where the pauper resides. The medical officer need not go further than common report, in his notice, either as to the alleged lunacy or as to the propriety of sending the party to an asylum. But it is otherwise with respect to lunatics not paupers and not wandering at large. There the informant states that the party "is deemed to be a lunatic," and "is not under proper care and control, or is cruelly treated or neglected," etc. Every relieving officer and every overseer of a parish of which there is no relieving officer, who respectively have knowledge, either by notice from a medical officer or otherwise, that any pauper resident within the district or parish is deemed to be a lunatic, shall within three days after obtaining such knowledge, give notice thereof to a justice having jurisdiction in the place where the pauper resides. A justice, upon receiving such notice, shall by order require the relieving officer or overseer giving the notice, to bring the alleged lunatic before him or some other justice having jurisdiction in the place where the pauper resides, at such time and place within three days from the time of the notice to the justice as shall be appointed by the order.

The justice before whom a pauper alleged to be a lunatic or an alleged lunatic wandering at large is brought shall call in a medical practitioner, and shall examine the alleged lunatic, and make such inquiries as he thinks advisable, and if the medical practitioner who is called in signs a medical certificate with regard to the lunatic, the justice may by order direct the lunatic to be received and detained in the institution for lunatics named in the order. Power is given to the justice to examine the alleged lunatic at his own house or elsewhere, in all respects as if the alleged lunatic had been brought before him. "At his own house" means the house of the alleged lunatic. The procedure under these sections is meant to be summary, and any person aggrieved by a justice's order has a right of appeal to quarter sessions.

A justice making an order for the reception of a lunatic otherwise than upon petition in the Lunacy Act of 1890, called a "summary reception order," may suspend the execution of the order for such period not exceeding 14 days, as he thinks fit. In urgent cases a constable, relieving officer, or overseer may remove an alleged lunatic to the workhouse, and the master of the workhouse shall, unless there is no proper accommodation in the workhouse for the alleged lunatic, receive the alleged lunatic therein, but no person shall be detained more than three days, and before the expiration of that time, such proceedings shall be taken with regard to the alleged lunatic as are required by the Act. If the master of the workhouse refuse to receive the lunatic on the ground of want of proper accommodation, steps should at once be taken to bring the lunatic before a justice who has power to send the lunatic to an asylum.

In any case where a summary reception order might be made, any justice, if satisfied that it is expedient for the welfare of the lunatic, or for the public safety, that the lunatic should forthwith be placed under care and control, and if it appears to him that there is proper accommodation for the lunatic in the workhouse, may make an order for receiving the lunatic in that workhouse. But the lunatic shall not be detained for more than fourteen days under this section. An order under this section (sect. 21) may be made by any justice having jurisdiction in the place where the lunatic is. In the case of a lunatic as to whom a summary reception order may be made, nothing in the Act shall prevent a relation or friend from retaining or taking the lunatic under his own care if a justice having jurisdiction to make the order, or the visitors of the asylum in which the lunatic is or is intended to be placed, shall be satisfied that proper care will be taken of the lunatic.

